# Regional variation in all-cause mortality among breast cancer patients in Germany: an analysis using the PopGrouper

**DOI:** 10.1007/s43999-026-00100-9

**Published:** 2026-11-05

**Authors:** Chrissa Tsatsaronis, Anika Kreutzberg, Reinhard Busse, Wilm Quentin

**Affiliations:** 1 Department of Health Care Management, Technische Universität Berlin, Berlin, Germany; 2 Department for Planetary and Public Health, Universität Bayreuth, Bayreuth, Germany

**Keywords:** Breast cancer, Mortality, Population classification, Regional variation, Risk adjustment, Germany

## Abstract

**Background:**

Breast cancer is the most common cancer among women in Germany. Although improvements in early detection and treatment have improved survival, differences in patient morbidity and healthcare delivery may contribute to regional variation in outcomes. All-cause mortality provides a measure that captures deaths related to both breast cancer and comorbid conditions. This study examines regional variation in all-cause mortality among breast cancer patients in Germany, using the PopGrouper as a novel morbidity adjustment tool.

**Methods:**

Claims data from the BARMER sickness fund for 2022–2023 were analyzed for actively treated breast cancer patients (*n* = 28,446). First, PopGroup-standardized observed-to-expected (O/E) ratios were calculated to describe regional variation of all-cause mortality in 2023, compared to the national average. Subsequently, the PopGrouper was used in multilevel regression analyses to analyze the association between age, morbidity, and all-cause mortality.

**Results:**

After PopGroup-standardization, O/E mortality ratios ranged between 0.4 and 2.0 between regions (coefficient of variation = 20%). The regression analysis did not confirm statistically significant differences in mortality between regions, but between age and morbidity groups.

**Conclusion:**

The PopGrouper can be used both for indirect standardization as in the O/E analyses and in regression models. Comparing both approaches can provide complementary perspectives and a more comprehensive assessment of regional variation. The PopGrouper represents a valuable tool for adjusting for morbidity-related care needs in regional comparative analyses.

**Supplementary Information:**

The online version contains supplementary material available at https://doi.org/10.1007/s43999-026-00100-9.

## Introduction

Breast cancer is the most common cancer in women in Germany [1] and places a considerable burden on those affected, their families, and the health care system [2–4]. For this reason, cancer prevention, early detection, and effective treatment have been high priorities on the public health agenda in Germany for several years [5]. Improvements in early detection and advances in treatment have significantly improved breast cancer survival. Despite the decline in mortality over recent decades [6], breast cancer remains one of the leading causes of death for women in Germany [7].As survival improves, many patients die from causes other than breast cancer, particularly at older ages and in the presence of multiple chronic conditions [8]. For this reason, all-cause mortality is an increasingly relevant outcome for health services research, as it reflects not only the prognosis of the cancer, but also the overall health status of patients and the quality of health care. Comorbidities are strong predictors for overall survival in patients with breast cancer and contribute to all-cause mortality [9]. All-cause mortality captures deaths related to comorbid conditions and provides a comprehensive measure for evaluating regional differences in healthcare outcomes among patients with breast cancer.

The incidence and mortality of breast cancer vary across the German federal states, with lower rates observed in the eastern states [6]. Previous studies have identified determinants of mortality among patients with breast cancer, including age and comorbidities such as HIV, diabetes, and obesity [10]. In health services research, observational studies commonly adjust for age and comorbidities to account for potential confounding [11]. Frequently applied measures include the Charlson Comorbidity Index [12] and the Elixhauser comorbidities [13]. Regional variation in mortality, after adjusting for age and morbidity, may reflect inequalities in access to care, differences in quality of care, or inefficiencies [14]. Therefore, identifying and explaining such regional variation is an important area of research.

In this paper, we explore variation in all-cause mortality among patients with breast cancer in Germany, adjusting for morbidity using the PopGrouper. The PopGrouper is a recently developed, population-based classification system that assigns individuals to mutually exclusive groups, accounting for multimorbidity and providing a high degree of morbidity differentiation [15]. Individuals within the same group have similar medical needs and healthcare costs, thereby enabling meaningful regional comparisons. For cancer, the PopGrouper differentiates between actively treated and non-actively treated malignant neoplasms and accounts for the presence of metastases [16].

The PopGrouper has demonstrated good predictive performance for mortality compared to the Charlson and Elixhauser comorbidity measures [17]. The use of the PopGrouper for regional comparisons was first introduced by Kreutzberg et al. [18] and subsequently tested in a diabetes population by Tsatsaronis et al. [19] and in a depression population by Kreutzberg et al. [20]. The present study extends this approach and demonstrates its application in a breast cancer population.

The overall objective of this paper is to test the PopGrouper’s potential for the assessment of regional health care variation. More specifically, this paper aims to explore regional variation in all-cause mortality among breast cancer patients and determine the extent to which regional differences are explained by differences in patient morbidity as measured by the PopGrouper.

## Methods

### Data and measures

#### Study population

We used pseudonymized claims data from the BARMER sickness fund for the years 2022 and 2023. BARMER covers approximately 10% of the German population (8.8 million in 2022) and operates in all German federal states. The data was accessed via the BARMER Scientific Data Warehouse, which contains information on demographics, inpatient and outpatient services, rehabilitation services, prescription drugs, remedies, and medical aids for all insured individuals. The analysis was conducted using SAS Enterprise Guide 8.4.

The study population included individuals aged 18 or older who were continuously insured by BARMER in 2022, and were either continuously insured in 2023 or deceased during that year. In addition, eligible individuals had to have a documented inpatient or outpatient diagnosis of breast cancer (ICD-10-GM: C50) and received active treatment in 2022. Active treatment was defined as either a hospitalization with breast cancer as the principal diagnosis or receipt of chemotherapy or radiotherapy (ICD-10-GM: Z51.0, Z51.1, Z51.2 or Z51.82).

The study population was restricted to actively treated cases because the total number of documented breast cancer diagnoses was several times higher than the known incidence in the population. This suggests that breast cancer diagnoses are not only recorded during episodes of active treatment but also over many subsequent years [16]. But still, given this definition, the study population included both incident and prevalent cases at various breast cancer stages. Individuals were excluded if they had missing or invalid ID, age, or postal code information or if they were assigned to the Macro PopGroup “Pregnancy, birth or postpartum”.

#### Outcome measure

The outcome measure was short-term all-cause mortality, defined as death from any cause in 2023.

#### Age, sex and PopGroups

Age was grouped into four categories (18–59, 60–69, 70–79, ≥ 80 years). Sex was defined as female or male/other. Multimorbidity was measured using the PopGrouper version 1.0, based on diagnoses, characteristics, and healthcare utilization in 2022 [15]. The PopGrouper comprises several hierarchical aggregation levels. At the highest level, individuals are assigned to one of 10 Macro PopGroups. These are further subdivided into 610 Base PopGroups, which provide much greater differentiation and reflect specific disease combinations and multimorbidity. Selected Base PopGroups are then further divided into more granular PopGroups, resulting in a total of 776 PopGroups. At each level of aggregation, individuals are assigned to exactly one group. Each PopGroup is assigned a composite severity score that reflects the relative complexity and severity of that group.

#### Regions

Each person was assigned to a region according to their place of residence. The 96 German spatial planning regions (Raumordnungsregionen) represented the regional unit of analysis [21]. Regions were classified into one of three levels of urbanization: urban region, region with intermediate urbanization, or rural region. This classification is based on the proportion of the population residing in large and medium-sized cities, the presence and size of large cities, overall population density, and population density excluding large cities [22]. In addition, each region was assigned a level of deprivation according to the German Index of Socioeconomic Deprivation (GISD). The most recent available version is based on data from 2019. The GISD measures relative regional socioeconomic deprivation and can range between 0 (lowest deprivation) and 1 (highest deprivation). Regions were subsequently grouped into five categories based on GISD quintiles [23].

### Analysis

The study population was described in terms of its demographic, socioeconomic, and PopGroup composition. The regional distribution of case rates and PopGroup severity in both populations was illustrated using maps.

The analytical approach proposed by Kreutzberg et al. [18] was applied and consisted of two components. First, PopGroup-standardized observed-to-expected (O/E) ratios were calculated. Subsequently, PopGroups were incorporated into regression analyses.

#### PopGroup-standardized observed-to-expected ratios

PopGroup-standardized observed-to-expected (O/E) ratios were calculated following the approach described by Kreutzberg et al. [18] to quantify regional variation in mortality among breast cancer patients. A PopGroup-standardized O/E mortality ratio compares the number of observed deaths in a population (e.g., a region) with the number of deaths that would be expected if that population experienced the same PopGroup-specific mortality rate as a reference population (e.g., the total national population). The O/E ratio therefore serves as a measure of relative regional performance compared to the national average, after standardizing for PopGroup composition (O/E = 1). An O/E ratio of 1.2 indicates that a region’s mortality among breast cancer patients is 20% higher than expected given the region’s PopGroup composition, while an O/E ratio of 0.8 indicates 20% lower mortality than expected. The PopGroup-standardized O/E ratios were compared to age-sex-standardized ratios to demonstrate differences in regional variation after adjusting for multimorbidity.

#### Regression analysis

Regression analyses were conducted to analyze the association between age, morbidity as measured by the PopGrouper, and mortality. A multilevel regression model using mixed-effects logistic regression with random intercepts at the regional level was specified to account for the hierarchical structure of the data, with patients clustered within regions, and mortality as the outcome.

In Model 1, only age groups (18–59, 60–69, 70–79, ≥ 80 years) were included as predictors. In Model 2, age groups and Base/Macro PopGroups were included. We chose to use Base PopGroups instead of PopGroups to avoid including groups with zero deaths, which could compromise the regression results. In addition, only Base PopGroups with a population size of at least 1% of the respective study population were included. For persons in Base PopGroups representing less than 1% of the study population, the higher-level Macro PopGroup was used instead.

## Results

### Descriptive results

#### Description of study population

In total, 28,525 BARMER insured individuals with actively treated breast cancer were identified. After excluding *n* = 79 individuals assigned to the Macro PopGroup “Pregnancy, birth and postpartum”, 28,446 individuals were included in the study population. Table 1 shows the study population’s demographic characteristics. The population was over 99% female and fairly evenly distributed across the four age groups. Nearly half resided in an urban region. More than one quarter resided in a region of medium deprivation according to the GISD, followed by one fifth in a region of second lowest deprivation, and another fifth in a region of lowest deprivation [23].

**Table 1:** Description of study population

	Study population	
	*N*	%
**Population size**	28,446	100.0
**Sex**		
Male	187	0.7
Female	28,259	99.3
**Age group**		
Age 18–59	7,957	28.0
Age 60–69	7,671	27.0
Age 70–79	7,413	26.1
Age > = 80	5,405	19.0
Level of urbanization*		
Urban region	13,523	47.5
Intermediate urbanization	8,296	29.2
Rural region	6,627	23.3
Level of deprivation**		
Lowest deprivation	5,420	19.1
Second lowest deprivation	5,675	20.0
Medium deprivation	7,627	26.8
Second highest deprivation	5,033	17.7
Highest deprivation	4,691	16.5
**Hospitalized**	11,587	40.7

Note: * 2022, ** 2019. Level of urbanization based on Federal Institute for Research on Building, Urban Affairs and Spatial Development [22]. Level of deprivation based on the German Index of Socioeconomic Deprivation (GISD) in 2019 [23]. Certified cancer centers certified by the German Cancer Society [24]

A total of 120 Base PopGroups and 154 PopGroups were present in the study population. The ten most frequent PopGroups, along with their severity scores, are listed in Table 2. The most frequent PopGroup (“Malignant neoplasm of the breast, actively treated + at most 1 (very) severe Major Disease Group and age ≥ 45 years”) accounted for a third of the total study population. The top ten PopGroups accounted for around three quarters of the study population. The majority of PopGroups are defined by the presence of a malignant neoplasm of the breast (or ductal carcinoma).

#### Description of outcome

In 2023, 7.5% (*n* = 2,119) of the study population died. Mortality varied considerably between PopGroups with a range from 0.0% to 20.2% for the ten most frequent PopGroups (Table 2). PopGroups with the highest severity score exhibited the highest mortality. Overall, mortality was lower in the top ten PopGroups than in the remaining less frequent ones, because the most severe PopGroups comprise fewer individuals. An equivalent table presenting the ten most frequent Base PopGroups is included in Supplementary Material 1.

**Table 2:** Top 10 PopGroups in the study population

Rank	PopGroup	PopGroup Description	*N*	%	Severity	Severity quintile	*N* deceased	% deceased
1	P04031BB	P04: Malignant neoplasm of the breast, actively treated + at most 1 (very) severe MDG and age ≥ 45 years	9,201	32.4	0.2	moderate	164	1.8
2	P04012ZB	P04: Malignant neoplasm of the breast with metastases, actively treated without novel therapy for malignant neoplasms	3,237	11.4	0.9	severe	305	9.4
3	P04025ZZ	P04: Ductal carcinoma in situ, actively treated	1,783	6.3	0.4	severe	12	0.7
4	P04027BB	P04: Neoplasms (MDG) & Neuromuscular disease + at most 1 (very) severe MDG and level of care dependency ≤ 1	1,688	5.9	0.3	moderate	21	1.2
5	P04031AZ	P04: Malignant neoplasm of the breast, actively treated + more than 1 (very) severe MDG	1,383	4.9	0.5	severe	52	3.8
6	P04023ZZ	P04: Malignant neoplasm of the breast, actively treated & Stress disorder or dissociative disorder	1,207	4.2	0.5	severe	24	2.0
7	P04012ZA	P04: Malignant neoplasm of the breast with metastases, actively treated with novel therapy for malignant neoplasms	945	3.3	1.3	severe	142	15.0
8	P04007ZB	P04: Malignant neoplasm of unclear site or neoplasms at multiple sites with metastases, actively treated without novel therapy for malignant neoplasms	747	2.6	1.5	very severe	151	20.2
9	P04031BA	P04: Malignant neoplasm of the breast, actively treated + at most 1 (very) severe MDG and age < 45 years	519	1.8	0.5	severe	0	0.0
10	P04027AZ	P04: Neoplasms (MDG) & Neuromuscular disease + more than 1 (very) severe MDG	463	1.6	0.7	severe	22	4.8
**Total Top 10 PopGroups**			**21**,**173**	**74.4**			**893**	**4.2**
**Total other PopGroups (*N*** **= 144)**			**7**,**273**	**25.6**			**1**,**226**	**16.9**

Note: Ranking by the number of individuals in each PopGroup, in descending order. The severity is measured using the PopGroup composite severity score, whereby a higher score indicates a higher severity. MDG: Major Disease Group. The MDGs are defined as part of the PopGrouper and classify the consolidated disease groups (CDGs) into mutually exclusive diagnosis areas. The level of care dependency classifies patients based on their dependency on long-term care and ranges from 0 (no care dependency) to 5 (highest level of care dependency) [25]. The first 3 digits of the PopGroup denote the overarching Macro PopGroup (MPG): P04: Actively treated malignant neoplasms. The severity quintile categorizes all 776 PopGroups into one of 5 categories (very severe, severe, moderate, mild, very mild)

#### Descriptive regional variation

**Fig. 1:**
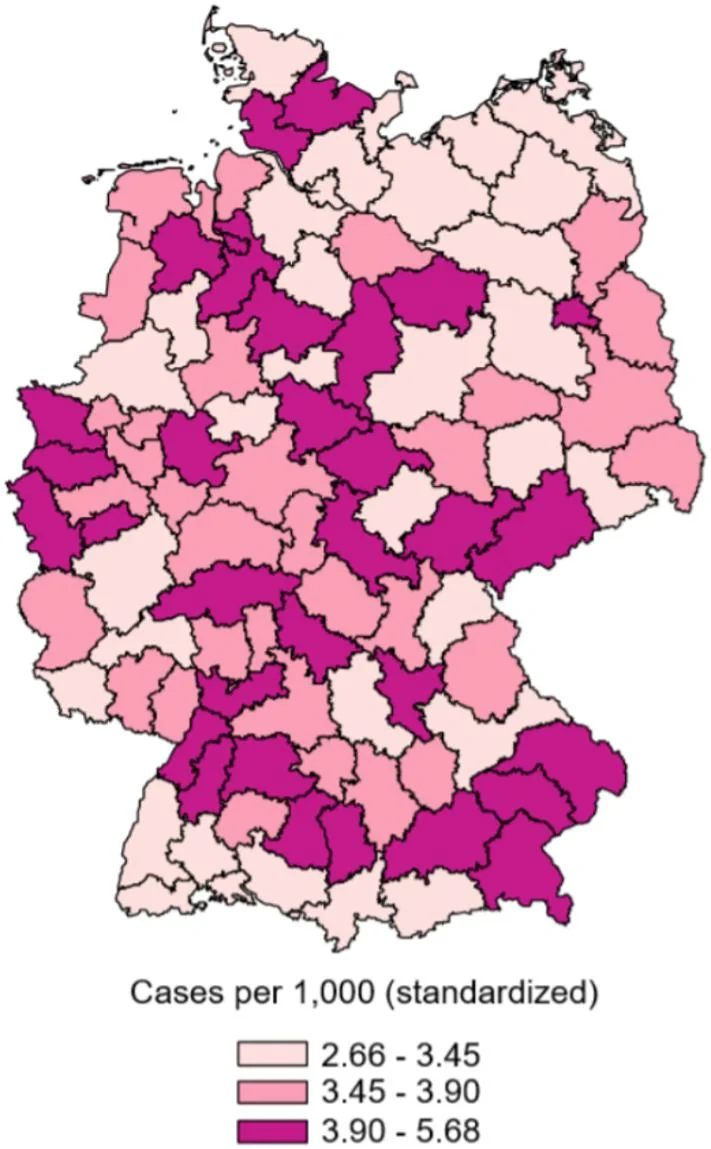
Breast cancer case rate per 1,000 BARMER insured individuals per region, age-sex-standardized

**Fig. 2:**
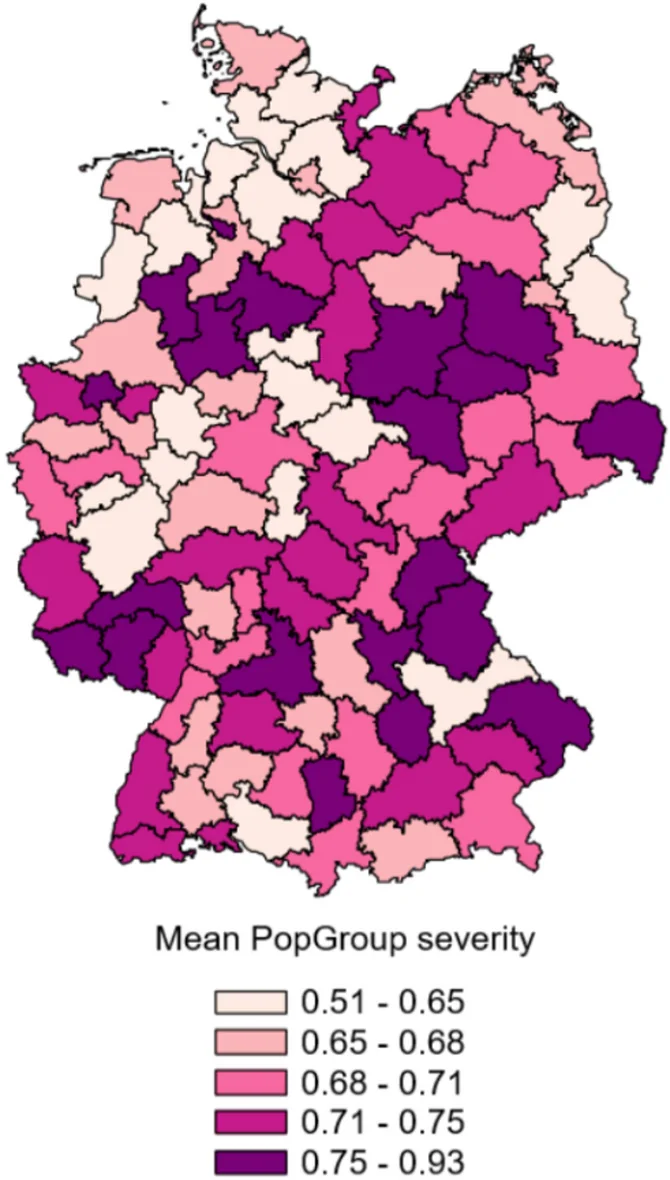
Average PopGroup severity level of breast cancer patients per region

On average, there were 296 individuals with breast cancer in each region, ranging from 52 to 1,493. Figure 1 visualizes the age-sex-standardized regional distribution of persons with breast cancer per 1,000 BARMER insured individuals in 2022, with darker regions indicating higher case rates. The case rate ranges from 2 to 6 persons with breast cancer per 1,000 insured. Overall, case rates were lower in the northeast (except for Berlin). Figure 2 shows the average PopGroup composite severity score of breast cancer patients per region. The severity score ranged between 0.51 and 0.93, with higher values indicating more severe PopGroups relative to all PopGroups in terms of mortality risk, health care utilization, and cost. The severity scores tended to be higher in the eastern federal states.

#### Regional variation in observed to expected outcome

**Fig. 3:**
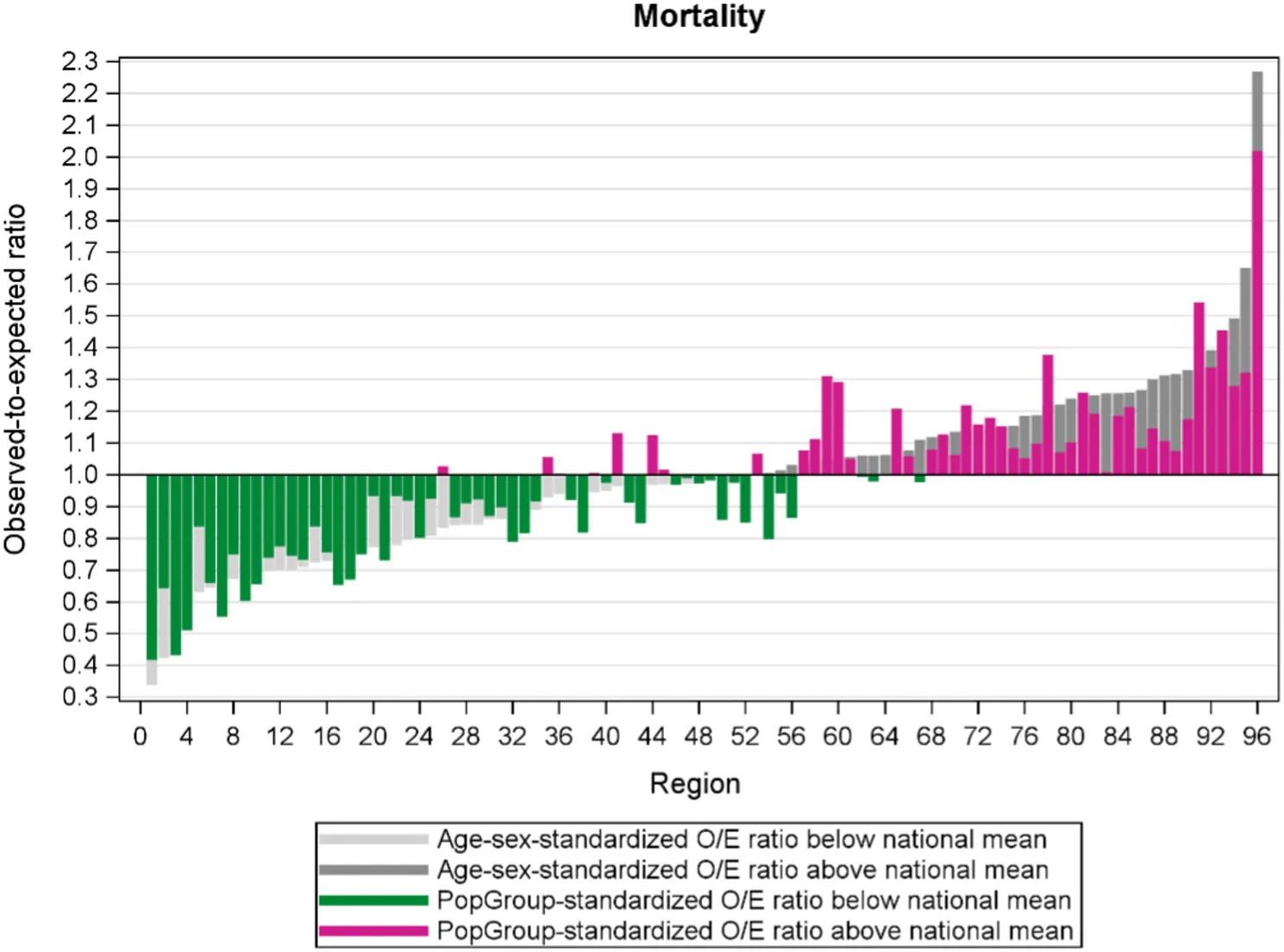
Age-sex-standardized and PopGroup-standardized mortality O/E ratios. Note: Regions are ranked by age-sex standardized O/E ratio. The reference line at 1.0 corresponds to the national mean. O/E: observed to expected ratio

**Fig. 4:**
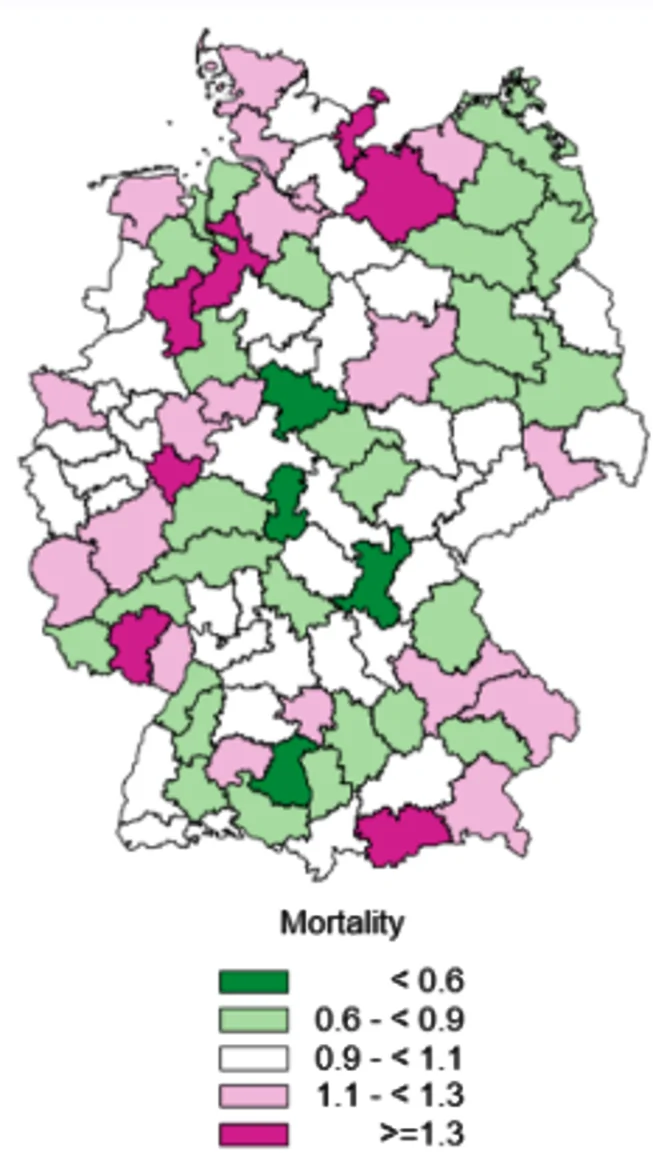
PopGroup-standardized mortality O/E ratios by region

Figure 3 shows the distribution of the age-sex-standardized and PopGroup-standardized mortality ratios for the 96 regions. Regions with a PopGroup-standardized mortality ratio above the national average (O/E > 1) are shown in pink, while regions below the national average (O/E < 1) are shown in green. The hidden light and dark grey bars represent the age-sex-standardized mortality ratio for each region. In each graph, the regions are ranked by the age-sex-standardized outcome ratios. This means that regions with a green bar but on the right side of the graph have moved from an above average to a below average value after PopGroup standardization, and vice versa. Table 3 shows measures of variation. The PopGroup-standardized O/E ratio ranges from 0.4 to 2.0 with a coefficient of variation of 20%, while the age-sex-standardized O/E ratio spans slightly wider from 0.3 to 2.3 with a coefficient of variation of 30%. Standardizing for morbidity using the PopGrouper therefore slightly reduced the relative regional variation in mortality. Figure 4 illustrates the spatial patterns of the PopGroup-standardized mortality ratio, with pink regions indicating mortality above and green regions mortality below average. There is no apparent regional pattern. A list of observed and expected mortality rates by region is included in Supplementary Material 2.

**Table 3:** Age-sex-standardized and PopGroup-standardized mortality O/E ratios

Outcome	Min	Max	Range	Mean	SD	CV
O/E mortality, PopGroup-standardized	0.4	2.0	1.6	1.0	0.2	20%
O/E mortality, age-sex-standardized	0.4	2.3	1.9	1.0	0.3	30%

Note: O/E: observed to expected ratio. Min: minimum, Max: maximum, SD: standard deviation, CV: coefficient of variation

### Regression results

Following the application of the 1% threshold for the regression analysis, 15 Base PopGroups and 2 aggregated Macro PopGroups were present in the population. The multilevel regression analysis with regional random effects revealed no statistically significant between region variation in mortality.

Table 4 presents the odds ratios and confidence intervals from the multilevel regression model. As expected, higher age is associated with higher mortality; patients aged 80 years or older have 3.6 times the odds of dying compared with patients aged 18–59. Several Base PopGroups are also significantly associated with higher mortality. Individuals assigned to the Base PopGroups P03051B and P03062B, both belonging to the Macro PopGroup P03 (Severe high-cost cases), have 19 to 20 times higher odds of dying, compared to the reference group (the most frequent Base PopGroup). Individuals assigned to the Base PopGroup P03061B (also belonging to the Macro PopGroup P03), P04007Z “Malignant neoplasm of unclear site or neoplasms at multiple sites with metastases, actively treated”, and the residual Macro PopGroup P03 have 14 to 15 times higher odds compared to the reference group.

**Table 4:** Multilevel regression results: odds ratios

Variable	Model 1				Model 2			
	OR	95% CI Lower	95% CI Upper	*p*-value	OR	95% CI Lower	95% CI Upper	*p*-value
Age 18–59	1.00	.	.	.	1.00	.	.	.
Age 60–69	1.50	1.29	1.75	< 0.0001	1.42	1.22	1.67	< 0.0001
Age 70–79	2.37	2.05	2.73	< 0.0001	1.97	1.70	2.29	< 0.0001
Age > = 80	4.50	3.91	5.17	< 0.0001	3.58	3.10	4.15	< 0.0001
P04031B: Malignant neoplasm of the breast, actively treated + at most 1 (very) severe MDG	.	.	.	.	1.00	.	.	.
P03051B: Aplastic anaemia or anaemia in neoplasms + at most 5 (very) severe MDGs	.	.	.	.	19.09	14.79	24.65	< 0.0001
P03062B: Severe pleural disease + at most 4 (very) severe MDGs	.	.	.	.	20.41	15.36	27.13	< 0.0001
P04007Z: Malignant neoplasm of unclear primary site or neoplasms at multiple sites with metastases, actively treated	.	.	.	.	15.33	12.42	18.93	< 0.0001
P03061B: Acute respiratory failure + at most 5 (very) severe MDGs	.	.	.	.	14.48	10.76	19.47	< 0.0001
P04012Z: Malignant neoplasm of the breast with metastases, actively treated	.	.	.	.	6.65	5.54	7.99	< 0.0001
P03053B: Complications following a procedure + at most 5 (very) severe MDGs	.	.	.	.	4.28	2.70	6.78	< 0.0001
P04027A: Neoplasms (MDG) & Neuromuscular disease + more than 1 (very) severe MDG	.	.	.	.	2.42	1.53	3.82	< 0.0001
P04020B: Chronic leukaemia or lymphoma, actively treated + at most 1 (very) severe MDG	.	.	.	.	2.37	1.37	4.08	< 0.0001
P04031A: Malignant neoplasm of the breast, actively treated + more than 1 (very) severe MDG	.	.	.	.	2.03	1.48	2.79	< 0.0001
P04026B: Malignant neoplasm of the female genital organs, actively treated + at most 1 (very) severe MDG	.	.	.	.	2.00	1.10	3.64	< 0.0001
P04030Z: Malignant neoplasm of unclear primary site or neoplasms at multiple sites, actively treated	.	.	.	.	1.74	0.93	3.24	< 0.0001
P04023Z: Malignant neoplasm of the breast, actively treated & Stress disorder or dissociative disorder	.	.	.	.	1.30	0.84	2.00	< 0.0001
P04027B: Neoplasms (MDG) & Neuromuscular disease + at most 1 (very) severe MDG	.	.	.	.	0.78	0.52	1.17	< 0.0001
P04025Z: Ductal carcinoma in situ, actively treated	.	.	.	.	0.44	0.24	0.78	< 0.0001
P03 “other”: Severe, high-cost cases*	.	.	.	.	15.73	13.08	18.92	< 0.0001
P04 “other”: Actively treated malignant neoplasms*	.	.	.	.	7.15	5.80	8.81	< 0.0001
N	28,446				28,446			

Note: OR: odds ratio, CI: Confidence interval, Model 1: adjustment for age group, Model 2: adjustment for age group, Base PopGroups/Macro PopGroups. Covariates with OR = 1 are the reference categories. Following the reference Base PopGroup, the remaining Base PopGroups are ordered by odds ratio in descending order. The first 3 digits of the Base PopGroup denote the overarching Macro PopGroup (MPG): P03: Severe, high-cost cases, P04: Actively treated malignant neoplasms. * The Macro PopGroups P03 and P04 in the last two lines include individuals from Base PopGroups representing less than 1% of the study population; these individuals were therefore grouped into residual Macro PopGroups to avoid exclusion

## Discussion

The present paper describes regional variation in breast cancer prevalence, PopGroups and all-cause mortality among breast cancer patients in Germany. The paper is part of a collection that presents and discusses the PopGrouper’s potential for assessing regional health care variation. It builds upon three previous publications that introduced the analytical framework [18] and presented an initial application in a diabetes population [19] and a depression population [20]. This work illustrates the utility of the PopGrouper as a tool for detailed risk adjustment in regional health care comparisons, using all-cause mortality among breast cancer patients in Germany as a case example.

Following the approach proposed by Kreutzberg et al. [18], PopGroups were used in two ways: first, to calculate morbidity-adjusted O/E ratios, and second, as morbidity covariates in regression analyses. The O/E analysis revealed regional variation in mortality among actively treated cancer patients, which may, however, be attributable to other factors that are not considered here.

The results of the O/E analysis are consistent with recent statistics which show lower age-standardized mortality rates in eastern federal states in Germany [6]. The observed variation was smaller with PopGroup standardization (CV: 20%) than with age-sex standardization (CV: 30%), suggesting that some of the variation may be explained by the morbidity groups.

Multilevel regression analysis did not confirm significant differences between regions after adjusting for age and PopGroup comorbidity. However, higher age and certain severe Base PopGroups were positively associated with all-cause mortality.

The apparent discrepancy between O/E ratio and regression results highlights some methodological considerations regarding the two approaches. The O/E ratios quantify how each region’s observed mortality compares to what would be expected given its case mix (in this case the PopGroup composition). This is a well-established and common approach to demonstrate regional variation in an easy to interpret manner [26]. However, it may overstate between-region differences, particularly in regions with smaller case numbers, where random fluctuation can produce extreme values. Particularly in the case of mortality, where the overall frequency of deaths is low, even large variations may reflect small absolute differences. There is therefore a risk of overestimating the true variation [27]. By contrast, the regression analyses are inferential models which explicitly account for individual-level variation and properly model uncertainty. They apply partial pooling (shrinkage) to region-level estimates, by which regions with smaller sample size or imprecise estimates are pulled toward the overall mean, reducing the influence of random noise [28]. At the same time, the small number of overall deaths may have limited the statistical power to detect between-region differences. As a result, regional variance may not reach statistical significance even when true differences exist. The fixed effects results are in line with prior research which found that higher age and comorbidities are associated with lower survival [10].

A key novelty of this paper is the use of the PopGrouper for risk-adjustment. The PopGrouper is comparable to existing international population classification systems [29, 30], but was specifically developed for the German health care context and is publicly available [15]. Unlike commonly used morbidity measures such as the Charlson and Elixhauser measures, PopGroups are mutually exclusive. This characteristic allows them to be applied both for indirect standardization (as in the O/E analyses), which are methodologically simpler and more transparent, and in regression models. Comparing both approaches can provide complementary perspectives and a more comprehensive assessment of regional variation.

In addition, the PopGrouper enables a differentiated measurement of morbidity. It integrates information from a broad range of health care services over an entire calendar year and accounts for the interactive effects of multimorbidity. The multilevel regression results demonstrate the value of this differentiation, as they revealed large variation in mortality risk across Base PopGroups, with more severe groups (based on the PopGrouper severity score) associated with significantly higher mortality.

Another advantage is the hierarchical structure of the PopGrouper, which includes Macro PopGroups, Base PopGroups and PopGroups, among others. This structure allows flexibility when selecting the appropriate aggregation level, depending on the application and required group size.

Some limitations should be considered. First, mortality is defined as all-cause mortality and not only specifically mortality due to breast cancer. It is important to keep this distinction in mind when comparing the results to statistics reporting breast cancer mortality rates.

Second, mortality was assessed over a one-year period. This relatively short follow-up may disproportionately capture deaths among patients at an advanced disease stage or patients transitioning to end-of-life care. Consequently, results reflect regional differences in short-term all-cause mortality and should not be interpreted as differences in long-term breast cancer survival or long-term quality of care.

Third, the O/E ratios assess regional performance relative to the national mean. This eliminates the need for a normative “optimal” benchmark and allows a focus on the extent of regional differences. However, this approach limits any claims regarding “good” or “bad” performance and only allows for relative terms such as “better” or “worse”, relative to the national average.

Fourth, the regression analysis focused specifically on age, Base PopGroups, and mortality, but did not account for additional potential confounders, such as disease stage or other cancer-specific clinical information (which is not available in claims data) or socioeconomic status. The aim was not to fully explain all predictors of mortality, but rather to examine these specific variables and test the PopGroupers application within this context.

Fifth, the use of data from a single sickness fund, which covers approximately 10% of the total German population, limits the representativeness and generalizability of the results. Although BARMER’s large size and nationwide presence enable analyses across all German regions, differences in demographic and morbidity structure compared with the general German population could influence prevalence and mortality estimates. For instance, the crude mortality rate among breast cancer patients appears to be slightly lower in the BARMER population than in the general German population [6]. The prevalence of actively treated breast cancer among the female BARMER population was around 560 per 100,000 in 2022. National statistics frequently report the incidence among women, which was around 180 per 100.000 in 2022 [6]. This implies that malignant neoplasms of the breast are actively treated for an average duration of 3 years.

Finally, the use of claims data comes with inherent limitations, including coding inaccuracies, upcoding, or underdiagnosis, which may result in deviations from true epidemiological data [31]. While the use of detailed selection and plausibility criteria – which are also used for the well-established risk-adjustment scheme among German sickness funds – helps mitigate the effects of upcoding, the possibility of underdiagnosis remains a limitation.

The aim of this research was to assess the applicability of the PopGrouper to describe regional variation in mortality among breast cancer patients in Germany. The analysis did not find statistically significant regional variation in mortality, which may be attributed in part to the small number of overall deaths. Overall, the study sought to demonstrate the PopGrouper’s potential contribution to the analysis of regional health care variation. It presents additional analytical possibilities for health systems research, beyond the conventional morbidity measures. In summary, the PopGrouper represents a valuable tool for adjusting for morbidity-related care needs in regional comparative analyses. Future research should build on these findings by using longer follow-up periods and larger populations to detect potential regional differences in relatively rare outcomes such as mortality, using the PopGrouper’s potential for morbidity-adjusted regional comparisons in breast cancer care.

## Supplementary Information

Below is the link to the electronic supplementary material.

Supplementary Material 1

Supplementary Material 2

## Data Availability

The data utilized in this current study are health insurance claims data provided by the German statutory health insurance fund BARMER. Due to legal and privacy restrictions, these data are not publicly available. Access to the data is subject to approval by the BARMER and may require additional agreements regarding data protection and confidentiality.
