# Supplementary material for: Regional variation in all-cause mortality among breast cancer patients in Germany: an analysis using the PopGrouper
Source: Res Health Serv Reg. 2026 Nov 5;5:17. doi: 10.1007/s43999-026-00100-9 (PMC13740209; doi:10.1007/s43999-026-00100-9)
Supplement: Supplementary file 1 [file 43999_2026_100_MOESM1_ESM.docx]

**Supplementary Material 1: Top 10 Base PopGroups in the study population**

| Rank | BPG | Description | N | % | N deceased | % deceased |
| --- | --- | --- | --- | --- | --- | --- |
| 1 | P04031B | P04: Malignant neoplasm of the breast, actively treated + at most 1 (very) severe MDG | 9,720 | 34.2 | 164 | 1.7% |
| 2 | P04012Z | P04: Malignant neoplasm of the breast with metastases, actively treated | 4,182 | 14.7 | 447 | 10.7% |
| 3 | P04027B | P04: Neoplasms (MDG) & Neuromuscular disease + at most 1 (very) severe MDG | 1,933 | 6.8 | 28 | 1.4% |
| 4 | P04025Z | P04: Ductal carcinoma in situ, actively treated | 1,783 | 6.3 | 12 | 0.7% |
| 5 | P04031A | P04: Malignant neoplasm of the breast, actively treated + more than 1 (very) severe MDG | 1,383 | 4.9 | 52 | 3.8% |
| 6 | P04023Z | P04: Malignant neoplasm of the breast, actively treated & Stress disorder or dissociative disorder | 1,207 | 4.2 | 24 | 2.0% |
| 7 | P04007Z | P04: Malignant neoplasm of unclear primary site or neoplasms at multiple sites with metastases, actively treated | 1,167 | 4.1 | 246 | 21.1% |
| 8 | P03051B | P03: Aplastic anaemia or anaemia in neoplasms + at most 5 (very) severe MDGs | 505 | 1.8 | 131 | 25.9% |
| 9 | P04027A | P04: Neoplasms (MDG) & Neuromuscular disease + more than 1 (very) severe MDG | 463 | 1.6 | 22 | 4.8% |
| 10 | P03053B | P03: Complications following a procedure + at most 5 (very) severe MDGs | 377 | 1.3 | 22 | 5.8% |
| Total Top 10 Base PopGroups | | | **22,720** | **79.9** | **1148** | **5.1%** |
| Total other Base PopGroups | | | **5,726** | **20.1** | **971** | **17.0%** |

Note: Ranking by the number of individuals in each Base PopGroup, in descending order. MDG: Major Disease Group. The MDGs are defined as part of the PopGrouper and classify the consolidated disease groups (CDGs) into mutually exclusive diagnosis areas. The level of care dependency classifies patients based on their dependency on long-term care and ranges from 0 (no care dependency) to 5 (highest level of care dependency). The first 3 digits of the Base PopGroup denote the overarching Macro PopGroup (MPG): P03: Severe, high-cost cases, P04: Actively treated malignant neoplasms.
