# Supplementary material for: Regional variation in all-cause mortality among breast cancer patients in Germany: an analysis using the PopGrouper
Source: Res Health Serv Reg. 2026 Nov 5;5:17. doi: 10.1007/s43999-026-00100-9 (PMC13740209; doi:10.1007/s43999-026-00100-9)
Supplement: Supplementary file 2 [file 43999_2026_100_MOESM2_ESM.docx]

**Supplementary Material 2: Observed and expected deaths by region**

| Region | N population | Observed mortality rate | N observed deaths | N expected deaths (PopGroup-standardized) | O/E |
| --- | --- | --- | --- | --- | --- |
| 1 | 434 | 6% | 26 | 27.8 | 0.9 |
| 2 | 189 | 9% | 17 | 14.0 | 1.2 |
| 3 | 181 | 12% | 21 | 14.4 | 1.5 |
| 4 | 353 | 6% | 21 | 22.9 | 0.9 |
| 5 | 112 | 7% | 8 | 7.1 | 1.1 |
| 6 | 471 | 8% | 40 | 33.1 | 1.2 |
| 7 | 582 | 8% | 45 | 47.8 | 0.9 |
| 8 | 129 | 11% | 14 | 9.1 | 1.5 |
| 9 | 71 | 4% | 3 | 3.6 | 0.8 |
| 10 | 153 | 7% | 11 | 11.2 | 1.0 |
| 11 | 224 | 4% | 8 | 15.6 | 0.5 |
| 12 | 151 | 7% | 11 | 9.9 | 1.1 |
| 13 | 510 | 8% | 42 | 42.3 | 1.0 |
| 14 | 173 | 6% | 11 | 11.9 | 0.9 |
| 15 | 119 | 8% | 9 | 8.9 | 1.0 |
| 16 | 197 | 6% | 11 | 13.1 | 0.8 |
| 17 | 163 | 17% | 28 | 13.9 | 2.0 |
| 18 | 265 | 8% | 22 | 19.1 | 1.2 |
| 19 | 65 | 6% | 4 | 5.1 | 0.8 |
| 20 | 106 | 8% | 8 | 9.4 | 0.9 |
| 21 | 619 | 8% | 48 | 45.7 | 1.1 |
| 22 | 247 | 7% | 17 | 15.0 | 1.1 |
| 23 | 430 | 6% | 26 | 35.6 | 0.7 |
| 24 | 511 | 8% | 40 | 37.1 | 1.1 |
| 25 | 478 | 6% | 30 | 29.2 | 1.0 |
| 26 | 389 | 7% | 29 | 29.8 | 1.0 |
| 27 | 901 | 10% | 88 | 74.2 | 1.2 |
| 28 | 1493 | 8% | 114 | 107.0 | 1.1 |
| 29 | 383 | 9% | 36 | 33.2 | 1.1 |
| 30 | 782 | 8% | 63 | 59.5 | 1.1 |
| 31 | 802 | 6% | 52 | 56.4 | 0.9 |
| 32 | 179 | 8% | 15 | 12.6 | 1.2 |
| 33 | 126 | 10% | 12 | 8.7 | 1.4 |
| 34 | 342 | 6% | 20 | 24.5 | 0.8 |
| 35 | 411 | 7% | 30 | 30.1 | 1.0 |
| 36 | 130 | 2% | 3 | 7.2 | 0.4 |
| 37 | 1021 | 7% | 69 | 79.3 | 0.9 |
| 38 | 484 | 7% | 33 | 32.8 | 1.0 |
| 39 | 452 | 8% | 38 | 32.2 | 1.2 |
| 40 | 261 | 7% | 19 | 22.0 | 0.9 |
| 41 | 281 | 11% | 30 | 23.5 | 1.3 |
| 42 | 256 | 9% | 22 | 20.0 | 1.1 |
| 43 | 143 | 11% | 16 | 12.1 | 1.3 |
| 44 | 79 | 5% | 4 | 5.4 | 0.8 |
| 45 | 60 | 3% | 2 | 4.6 | 0.4 |
| 46 | 125 | 10% | 12 | 11.9 | 1.0 |
| 47 | 128 | 9% | 11 | 10.0 | 1.1 |
| 48 | 337 | 5% | 17 | 25.9 | 0.7 |
| 49 | 72 | 8% | 6 | 5.2 | 1.2 |
| 50 | 156 | 5% | 8 | 10.3 | 0.8 |
| 51 | 104 | 9% | 9 | 7.7 | 1.2 |
| 52 | 57 | 5% | 3 | 5.0 | 0.6 |
| 53 | 413 | 8% | 32 | 32.8 | 1.0 |
| 54 | 326 | 7% | 23 | 23.6 | 1.0 |
| 55 | 474 | 7% | 33 | 31.2 | 1.1 |
| 56 | 101 | 7% | 7 | 7.7 | 0.9 |
| 57 | 264 | 6% | 16 | 18.5 | 0.9 |
| 58 | 83 | 8% | 7 | 7.2 | 1.0 |
| 59 | 109 | 7% | 8 | 9.3 | 0.9 |
| 60 | 202 | 10% | 20 | 17.5 | 1.2 |
| 61 | 452 | 9% | 40 | 37.7 | 1.1 |
| 62 | 52 | 6% | 3 | 4.0 | 0.8 |
| 63 | 130 | 5% | 7 | 10.7 | 0.7 |
| 64 | 216 | 8% | 17 | 15.9 | 1.1 |
| 65 | 873 | 8% | 66 | 66.7 | 1.0 |
| 66 | 95 | 9% | 9 | 8.4 | 1.1 |
| 67 | 187 | 4% | 8 | 14.5 | 0.6 |
| 68 | 94 | 9% | 8 | 6.1 | 1.3 |
| 69 | 142 | 6% | 8 | 12.0 | 0.7 |
| 70 | 106 | 8% | 8 | 6.6 | 1.2 |
| 71 | 320 | 8% | 27 | 24.0 | 1.1 |
| 72 | 90 | 7% | 6 | 6.6 | 0.9 |
| 73 | 183 | 5% | 10 | 12.5 | 0.8 |
| 74 | 319 | 7% | 22 | 27.6 | 0.8 |
| 75 | 1324 | 8% | 105 | 97.5 | 1.1 |
| 76 | 427 | 7% | 29 | 35.5 | 0.8 |
| 77 | 321 | 5% | 16 | 21.6 | 0.7 |
| 78 | 261 | 6% | 15 | 16.3 | 0.9 |
| 79 | 215 | 7% | 14 | 16.5 | 0.9 |
| 80 | 190 | 5% | 10 | 13.3 | 0.8 |
| 81 | 133 | 5% | 6 | 9.1 | 0.7 |
| 82 | 178 | 10% | 17 | 13.5 | 1.3 |
| 83 | 236 | 5% | 11 | 14.7 | 0.8 |
| 84 | 232 | 10% | 24 | 18.0 | 1.3 |
| 85 | 242 | 10% | 23 | 17.8 | 1.3 |
| 86 | 169 | 9% | 16 | 15.2 | 1.1 |
| 87 | 490 | 7% | 32 | 34.3 | 0.9 |
| 88 | 230 | 8% | 18 | 17.9 | 1.0 |
| 89 | 77 | 8% | 6 | 5.5 | 1.1 |
| 90 | 129 | 6% | 8 | 8.9 | 0.9 |
| 91 | 277 | 7% | 20 | 20.7 | 1.0 |
| 92 | 376 | 9% | 34 | 30.7 | 1.1 |
| 93 | 207 | 6% | 12 | 16.4 | 0.7 |
| 94 | 192 | 3% | 6 | 9.3 | 0.6 |
| 95 | 217 | 7% | 15 | 16.4 | 0.9 |
| 96 | 205 | 7% | 15 | 15.3 | 1.0 |
| Total | **28,446** | **7%** | **2,119** | **2,119** | **1** |

Note: “Region” represents one of 96 spatial planning regions in Germany – numbers are randomly allocated. “Observed mortality” is defined as all-cause mortality in the year 2023. “N expected deaths” = number of expected deaths in the region given the regional PopGroup distribution, i.e., for a given region the sum of the number of patients in each PopGroup multiplied by the PopGroup-specific national average mortality. “O/E” = PopGroup standardized observed-to-expected mortality ratio, i.e., N observed deaths divided by N expected deaths. Ratios > 1 reflect regional standardized mortality above the national average and ratios < 1 below the national average. For a formal presentation please refer to Kreutzberg et al. (2025).

Kreutzberg A, Tsatsaronis C, Grobe TG, et al (2025) The PopGrouper as a tool for morbidity adjustment in regional comparisons of health care: an analytical framework. Research in Health Services & Regions 4:. https://doi.org/10.1007/s43999-025-00068-y
